# Blood culture-negative infective endocarditis: are we looking hard enough?

**DOI:** 10.1007/s15010-023-02097-6

**Published:** 2023-10-04

**Authors:** Frazer Kirk, Natasha Marcella Vaselli

**Affiliations:** 1 Gold Coast University Hospital, Southport, QLD Australia; 2 School of Medicine and Dentistry, Griffith University, Gold Coast, QLD Australia; 3 Institute of Infection, Veterinary and Ecological Sciences, University of Liverpool, Liverpool, UK; 4 NIHR Health Protection Research Unit in Gastrointestinal Infections at the University of Liverpool, Liverpool, UK

**Keywords:** Blood culture negative endocarditis, Literature review, Infective endocarditis

## Abstract

**Introduction:**

Infective endocarditis is a common cardiac condition, with significant mortality. Blood culture-negative endocarditis is an important subgroup of endocarditis that holds significant morbidity and mortality.

**Method:**

We performed an updated review of the literature. We searched the databases of Web of Science, MEDLINE, EMBAS and Scopus for the latest clinical guidelines and literature on blood culture negative endocarditis to provide a narrative synthesis of the literature.

**Results:**

There is significant heterogeneity in causes and complications of culture-negative infective endocarditis, due to an insensitivity in available clinical diagnostic pathways. Despite significant advances in diagnostic tools, the diagnostic criterion for infective endocarditis (the modified Duke’s criterion) remains insensitive to the detection of culture-negative infective endocarditis.

**Conclusion:**

The natural history of BCNE and our diagnostic resources are changing. It is time our criterion did too. Remembering, BCNE holds significant morbidity and mortality—the absence of organism of culture should not reassure, rather concern clinicians. Every effort should be made to accurately identify organisms.

## Introduction

Infective endocarditis (IE) is a common life-threatening cardiac condition with a prevalence of 5–14.3/100,000 adults per year [1] and IE carries a significant in-hospital mortality of between 6 and 50% [2]. Wide prevalence and mortality reflect that IE is a heterogeneous pathology that encompasses a wide range sub-types of endocarditis, importantly blood culture-negative endocarditis (BCNE), which represents between 2.5 and 31% of all IE presentations [3]. BCNE remains a diagnostic challenge despite advances in diagnostic techniques. It continues to be associated with significantly higher in-hospital and long-term mortality, compared to its counterpart—blood culture-positive endocarditis (BCPE) [4].

The most common cause of BCNE is due to the early initiation of antibiotics prior to culture and the organisms responsible for this reflect that of BCPE. “True” BCNE is due to intra-cellular bacteria that cannot be routinely cultured using blood [5]. These organisms include *Coxiella burnetii* (Q fever), *Bartonella* spp*., Brucella* spp*.*, *Tropheryma whipplei, Mycobacteria* species and non-*Candida* fungi [5, 6].

The modified Duke’s criterion is a scoring system used to aid clinicians in the diagnostic classification of IE. The sensitivity of the modified Duke’s criterion to provide a definitive diagnosis of IE has been reported as approximately 70% in cardiac devices and 80% in native valves [2]. However, the sensitivity of the criterion is diminished in BCNE [6]. One case series in the UK showed that the modified Duke’s criterion performed poorly among BCNE and they recommend the addition of the St Thomas’ minor criteria to allow a more definitive diagnosis among blood culture-negative cases. They report that the use of the modified Duke’s criterion diagnosed only 32% of proven native valve cases as definite, compared to 64% when the St Thomas’ modifications were used [6].

Over the last 20 years, there has been many advances made in the diagnosis of BCNE. Increasingly, clinicians are utilizing molecular testing and polymerase chain reaction (PCR) to identify causative organisms in IE, such as 16S rRNA PCR, a broad-range bacterial PCR. It is recommended that PCR is performed on valve tissue and can increase the diagnostic yield, successfully identifying organisms in 60–100% of cases [7]. Meta-genomic Next-Generation Sequencing (mNGS), is an involving technology with increasing importance in the diagnosis and management of IE [8]. mNGS boasts a wider diagnostic spectrum and may be helpful as a marker of microbial killing. However, despite the benefits of this technology, it is not widely available in less-affluent countries. Consequently, inclusion of nMGS in the diagnostic criteria for IE in the contemporary climate may not change the natural history or identification of BCNE for a great proportion of the population.

With the advancement of testing techniques comes the need for updated criteria to reflect these changes. Currently, the modified Duke’s criterion is used to aid clinicians in the diagnosis of IE, with criteria addressing serological assessment for *Coxiella*. However, it does not factor in serological assessment for *Bartonella* spp. or the use of PCR. An updated criterion for BCNE may help with earlier identification and decrease morbidity and mortality associated with the condition.

The European Society of Cardiology, has recognized the difficulties in the diagnosis of BCNE, and suggested their own pathway for the investigation of BCNE, which takes a multi-modal clinical diagnostic approach, using complimentary clinical assessment, culture/serology and diagnostic imaging [9]. This guideline is very comprehensive and includes the main causative organisms of BCNE, which are not reflected in the modified Duke’s criterion. Incorporation of this into the modified Dukes to include serology and PCR of other common culture-negative organisms may help improve detection and appropriate treatment of BCNE.

However, this guideline needs to be updated to include the advances in molecular testing such as mNGS, in which studies have shown to be superior to other diagnostic techniques [8]. By incorporating updated molecular techniques, this may lead to quicker time of identification of the causative organism, leading to correct management and improved clinical outcomes.

With the changes in global health care over time, we are seeing an aging population, increasing rates of implanted cardiac devices, and consequently an increasing number of cardiac infections [10]. The natural history of IE, particularly BCNE is changing, and our diagnostic criteria need to reflect this.

## Conclusion

Despite evolving technology improving the identification of infective organism, the modified Duke’s criterion for diagnosis of IE remains insensitive to the detection of BCNE. The natural history of BCNE and our diagnostic resources are changing. It is time our criterion did too. Remembering, BCNE holds significant morbidity and mortality—the absence of organism of culture should not reassure, rather concern clinicians. Every effort should be made to accurately identify organisms.
